# Reduced acquisition time PET pharmacokinetic modelling using simultaneous ASL–MRI: proof of concept

**DOI:** 10.1177/0271678X18797343

**Published:** 2018-09-05

**Authors:** Catherine J Scott, Jieqing Jiao, Andrew Melbourne, Ninon Burgos, David M Cash, Enrico De Vita, Pawel J Markiewicz, Antoinette O'Connor, David L Thomas, Philip SJ Weston, Jonathan M Schott, Brian F Hutton, Sébastien Ourselin

**Affiliations:** 1 Translational Imaging Group, CMIC, University College London, London, UK; 2 Inria, Aramis project-team, Institut du Cerveau et de la Moelle épinière, Inserm, CNRS, Sorbonne Université, Paris, France; 3 Dementia Research Centre, Institute of Neurology, University College London, London, UK; 4 Neuroradiological Academic Unit, UCL Institute of Neurology, London, UK; 5 Lysholm Department of Neuroradiology, National Hospital for Neurology and Neurosurgery, UCL Hospitals Foundation Trust, London, UK; 6 Department of Biomedical Engineering, School of Biomedical Engineering & Imaging Sciences, King's College London, King's Health Partners, St Thomas' Hospital, London, UK; 7 Leonard Wolfson Experimental Neurology Centre, UCL Institute of Neurology London, UK; 8 Institute of Nuclear Medicine, University College London, London, UK; 9 Centre for Medical Radiation Physics, University of Wollongong, NSW, Australia; 10 School of Biomedical Engineering & Imaging Sciences, King's College London, London, UK

**Keywords:** Positron emission tomography, arterial spin labelling, pharmacokinetic modelling, reduced acquisition time, cerebral blood flow

## Abstract

Pharmacokinetic modelling on dynamic positron emission tomography (PET) data is a quantitative technique. However, the long acquisition time is prohibitive for routine clinical use. Instead, the semi-quantitative standardised uptake value ratio (SUVR) from a shorter static acquisition is used, despite its sensitivity to blood flow confounding longitudinal analysis. A method has been proposed to reduce the dynamic acquisition time for quantification by incorporating cerebral blood flow (CBF) information from arterial spin labelling (ASL) magnetic resonance imaging (MRI) into the pharmacokinetic modelling. In this work, we optimise and validate this framework for a study of ageing and preclinical Alzheimer's disease. This methodology adapts the simplified reference tissue model (SRTM) for a reduced acquisition time (RT-SRTM) and is applied to [^18^F]-florbetapir PET data for amyloid-β quantification. Evaluation shows that the optimised RT-SRTM can achieve amyloid burden estimation from a 30-min PET/MR acquisition which is comparable with the gold standard SRTM applied to 60 min of PET data. Conversely, SUVR showed a significantly higher error and bias, and a statistically significant correlation with tracer delivery due to the influence of blood flow. The optimised RT-SRTM produced amyloid burden estimates which were uncorrelated with tracer delivery indicating its suitability for longitudinal studies.

## Introduction

Positron emission tomography (PET) facilitates the quantification of a range of important biomarkers through the injection and detection of targeted radiotracers. To interpret the measured signal and derive the biological parameters of interest, data are collected dynamically from injection, covering radiotracer delivery to tissue, interaction with the target, and tracer washout. This provides a map of the spatio-temporal concentration of the tracer *in vivo*. A pharmacokinetic model which describes these processes may then be fitted to these dynamic data to estimate the biological parameters such as radiotracer target density.

Depending on the radiotracer administered, the dynamic data acquisition time required to fit the model may be 60 min or more. This is prohibitive in a clinical context due to patient discomfort, restrictions on scanner time availability, and the increased chance of subject motion which corrupts the data. Consequently, a simplified technique is commonly employed.

The standardised uptake value ratio (SUVR) is a measure of relative tracer uptake which can be calculated from a static scan lasting approximately 10 min. SUVR is calculated by dividing the tracer concentration within the tissue of interest by the concentration in a reference region. The reference region consists of tissue considered to be free of the radiotracer target and represents the non-displaceable (ND) tracer concentration (i.e. tracer in the tissue which is not bound to the intended target). When the ratio of the tracer concentration within the target tissue and the reference tissue has reached a steady-state, SUVR approximates the distribution volume ratio (DVR). DVR can be estimated from pharmacokinetic modelling and is related to target density.

However, as SUVR is calculated from a single static scan, the tracer concentration present during the acquisition will depend on the delivery of the tracer, as well as target density. Tracer delivery is intrinsically linked to blood flow, and since blood flow can change during the progression of disease,^1^ and indeed fluctuate over the course of a day,^2^ SUVR estimates may be confounded.

The influence of cerebral blood flow (CBF) changes on SUVR estimates has been highlighted in longitudinal Alzheimer's disease studies in which the target of interest was the protein amyloid-β. Amyloid-β is an early indicator of disease onset and a therapeutic target, hence accurately quantifying amyloid-β density is of paramount importance. Here, variation in blood flow has been shown to cause spurious changes in SUVR which are unrelated to target density.^3,4^ Conversely, target density estimates derived from pharmacokinetic modelling of dynamic data starting from radiotracer injection can account for blood flow, as tracer delivery is parameterised within the model.

Dynamic PET data can be divided into two phases: the early phase, in which the signal is dominated by tracer delivery to tissue,^5^ and the late phase, which contains information related to tracer binding and washout, and is where SUVR is estimated. The intrinsic correlation between tracer delivery and CBF has been demonstrated for an amyloid-β tracer.^6^ Therefore, if CBF can be measured independently from the PET acquisition, then the data acquisition time may be reduced such that only the late phase data are acquired to estimate the remaining parameters.

Arterial spin labelling (ASL) magnetic resonance imaging (MRI) is a non-invasive imaging technique which applies a magnetic ‘tag’ to arterial blood, such that it can be used as an endogenous contrast agent. ASL can be used to estimate CBF, as validated by comparison with the gold standard radiolabelled water PET.^7^ While the accuracy of the technique is dependent on the implementation, high quantitative accuracy has been achieved when ASL data are normalised to a reference region.^8^

The introduction of combined PET/MRI scanners, which facilitate simultaneous acquisition, means that ASL and late phase PET data can be acquired concurrently. By incorporating CBF information from ASL into the PET pharmacokinetic modelling to provide early phase delivery information, the total acquisition time can be significantly reduced, increasing patient comfort and throughput, without sacrificing quantitative accuracy.

In this paper, we build on the framework which combines ASL-derived CBF data with PET kinetic modelling to reduce the acquisition time from 60 to 30 min.^9^ This is referred to here as the reduced acquisition time simplified reference tissue model (RT-SRTM). In the present work, the relationship between PET radiotracer delivery and ASL measured CBF is investigated with a larger group of subjects. An improved methodology for the estimation of the early phase data is proposed and compared to that used previously.^9^ Finally, the timing of the late phase acquisition is optimised before applying the improved methodology to a different set of subjects. This was applied to [^18^F]-florbetapir amyloid PET data from a study of ageing and preclinical Alzheimer's disease.

## Material and methods

### PET kinetic modelling

The simplified reference tissue model (SRTM)^10^ was used as the gold standard for PET pharmacokinetic modelling, as it is commonly applied in amyloid studies^5,6,11,12^ and has been validated against pharmacokinetic modelling with arterial sampling for [^18^F]-florbetapir.^13^ The SRTM employs a reference region, which is considered to be devoid of the imaging target, to replace the plasma input function. Cerebellar grey matter was used as it is assumed to be devoid of amyloid-β.^14^

The operational equation between the tracer concentration in the target tissue $C_{T}(t)$ and the reference region $C_{R}(t)$ is formulated as shown in equation (1). Here *t* denotes time with tracer injection at t=0, and ⊗ represents the convolution operator

(1) $$C_{T}(t)=R_{1}C_{R}(t)+(k_{2}-R_{1}\frac{k_{2}}{1+{\mathrm{BP}}_{\mathrm{ND}}})C_{R}(t)⊗e^{\frac{-k_{2}}{1+{\mathrm{BP}}_{\mathrm{ND}}\;}t}$$

The SRTM contains three parameters: *R*_1_, which is the rate constant of tracer delivery to the target tissue relative to the reference tissue; *k*_2_, which is the rate constant from target tissue to blood; and the *ND* binding potential ${\mathrm{BP}}_{\mathrm{ND}}$, which is proportional to target density, i.e. the density of amyloid-β, see Supplementary Materials.

An in-house implementation of the SRTM using the basis function method^15^ was fitted to the PET data to derive regional gold standard parameter estimates, denoted by an asterisk, of ${\mathrm{BP}}_{\mathrm{ND}}^{*}$, $k_{2}^{*}$ and $R_{1}^{*}$ from dynamic PET data conventionally acquired for t∈[0,60] min, denoted here as t=0,60 min. For more details see Supplementary Materials.

### PET kinetic modelling with reduced acquisition time

Early PET signal is dominated by the delivery of the tracer to the tissue and hence is important for the estimation of *R*_1_. The later part of the signal contains information about the binding of the tracer to the target and its subsequent washout, which is essential for the estimation of *k*_2_ and ${\mathrm{BP}}_{\mathrm{ND}}$.

Consequently, if the blood flow information can be estimated independently from the PET data, then the acquisition time can be reduced, by recording only the late signal to allow the estimation of *k*_2_ and ${\mathrm{BP}}_{\mathrm{ND}}$. This requires two modifications to the model: (i) the estimation of *R*_1_ from another source, and (ii) extrapolation of the reference region curve, *C_R_* to t=0, as the model contains a convolution term which requires the full time series from injection to compute. Here, we refer to this modified model as the reduced acquisition time SRTM (RT-SRTM).

### Derivation of PET-*R*_1_ from ASL–CBF

*R*_1_ is defined as R1=K1/K1' where *K*_1_ is the transfer rate constant from blood to target tissue and K1' is the transfer rate constant from blood to reference tissue. According to the Renkin–Crone^16,17^ capillary model, the relationship between tracer delivery, *K*_1_, and blood flow, *F*, can be described as

(2) $$K_{1}=\mathrm{EF}=(1-e^{\frac{-\mathrm{PS}}{F}})F$$

The Renkin–Crone model includes a term for the net extraction of the tracer from the capillaries, *E*, which is dependent on the vessel permeability surface area product, PS. Under common physiological flow conditions, where PS/F is high (≥3), the relationship between *K*_1_ and flow *F* is approximately linear. If we assume that PS is sufficiently high, the relationship between *K*_1_ and *F*, and in turn the relationship between *R*_1_ and *F*, can be approximated as a linear function,^6^ as expressed in equation (3)

(3) R1=K1K1'=β0+β1FF'

ASL can be used to measure the CBF, F, and may be converted into an *R*_1_ estimate to use in the RT-SRTM, using the relationship from equation (3), where F' indicates the CBF measured in the reference region.

In this study, linear regression between *R*_1_ and CBF was performed in a group of subjects to determine whether this approximation is valid. The slope and intercept of the linear regression, $\beta_{0}$ and $\beta_{1}$, can account for systematic differences between the modalities and an extraction fraction of E<100%. The derived $\beta_{0}$ and $\beta_{1}$ can then be applied to a different group of subjects to convert ASL-CBF to a derived *R*_1_ value.

Due to systematic errors in ASL-CBF estimates in certain regions of the brain, particularly for single inversion time ASL data,^7^ multi-linear analysis was also performed, to determine whether $\beta_{0}$ and $\beta_{1}$ may be region dependent. Multi-linear regression has the capacity to model interaction terms between ASL-CBF and the region and can be described as in equation (4)

(4) $$\begin{array}{c} R_{1}=\beta_{0}+\beta_{1}\frac{F}{F'}+\sum_{n=2}^{\mathrm{regions}}\beta_{n}I[{\mathrm{region}}_{n}]\\ +\sum_{m=\mathrm{regions}+1}^{2\times \mathrm{regions}}\beta_{m}\times \frac{F}{F'}\times I[{\mathrm{region}}_{m}] \end{array}$$

Here $[{\mathrm{region}}_{n}]$ is equal to 1 when ${\mathrm{region}}_{n}$ is being considered, and 0 otherwise. Two further multi-linear analyses were also performed (i) using the subject as a covariate, and (ii) using both subject and regions as covariates to determine their relative influence on *R*_1_ estimation.

#### Extrapolation of PET reference region, $C_{R(t)}$

To compute the convolution term in equation (1), *C_R_* must be known from injection, at t=0, to the end of the scan, $t=t_{e}$, where $t_{s},t_{e}=0,60$ min for the gold standard. However, when reducing the acquisition time, *C_R_* is only measured between *t_s_* and $t_{e}$ where $t_{s}\neq 0$, and therefore a strategy is required to extrapolate the missing data. Here we define $C_{R}(t)$ as a vector containing the reference region tracer concentration over time, $C_{R}^{\mathrm{pop}}(t)$ as a matrix containing the reference region concentration for a population of subjects, and ${\bar{C}}_{\text{R}(\text{t})}^{\text{pop}}$ as a vector containing the mean population tracer concentration. For clarity, the acquisition time, *t*, is expressed as a discrete variable, as the dynamic data are binned into frames.

In this work, two different approaches are evaluated to estimate the whole reference input vector $C_{[t=0,t_{e}]}$ for an unseen subject's $C_{[t=t_{s},t_{e}]}$. Both techniques make use of a population of subjects for which the full $C_{[t=0,t_{e}]}$ was measured.

The first method, referred to as the *scaled mean* *C_R_* method and proposed by Scott et al.,^9^ scales the population average reference region curve, ${\bar{C}}_{\text{R}[\text{t}=0,{\text{t}}_{\text{e}}]}^{\text{pop}}$, as

(5) $$C_{R[t=0,t_{e}]}\approx \alpha {\bar{C}}_{R[t=0,t_{e}]}^{\mathrm{pop}}$$

where α is a subject specific scaling factor determined through a least squares fit of ${\bar{C}}_{\text{R}[\text{t}={\text{t}}_{\text{s}},{\text{t}}_{\text{e}}]}^{\text{pop}}$ to $C_{R[t=t_{s},t_{e}]}$. This results in an individual estimate of $C_{R[t=0,t_{e}]}$ to be used in the RT-SRTM.

The second method employs statistical shape modelling to build a model of the variation in *C_R_* within the population of subjects.^18^ This requires principal component analysis (PCA) of a set of subjects to determine the *M* components, $U=[u_{1},\ldots ,u_{M}]'$ where $u_{i}=[u_{1},\ldots ,u_{\mathrm{frames}}]$, and is therefore referred to as the *PCA* *C_R_* method.

The $C_{[t=0,t_{e}]}$ of each subject in the set can be expressed as the mean population reference curve, ${\bar{C}}_{\text{R}[\text{t}=0,{\text{t}}_{\text{e}}]}^{\text{pop}}$, plus a linear combination of the weighted principal components, where the weight of the ith mode, $u_{i}$, is *w_i_*. A subset, *L* where L<M, of the components which describe the majority of the variation are selected. An unseen reference region curve $C_{R_{[t=t_{s},t_{e}]}}$ can then be fitted by adjusting the weights, as per equation (6). The same weights and modes can then be used with $C_{R_{[t=0,t_{e}]}}^{\mathrm{pop}}$ to generate $C_{R_{[t=0,t_{e}]}}$. See Supplementary Materials for more details

(6) $${\text{C}}_{{\text{R}}_{[\text{t}={\text{t}}_{\text{s}},{\text{t}}_{\text{e}}]}}\approx {\bar{C}}_{\text{R}[\text{t}={\text{t}}_{\text{s}},{\text{t}}_{\text{e}}]}^{\text{pop}}+\sum_{i=1}^{L}w_{i}u_{i}$$

#### Fitting the SRTM with CBF-derived *R*_1_ and extrapolated *C_R_*

To apply the modifications to the SRTM with reduced acquisition time, the operational equation in (1) is re-written as in equation (7). This groups the measured parameter $C_{T}(t)$, with the derived $C_{R}(t)$ and derived *R*_1_ into a dummy variable, $C_{T}^{d†}(t)$, as they are determined prior to fitting

$$C_{T}^{d†}(t)=C_{T}(t)-R_{1}C_{R}(t)=\varphi C_{R}(t)⊗e^{-\theta t}$$

where

(7) $$\varphi =k_{2}-R_{1}\frac{k_{2}}{\left(1+{\mathrm{BP}}_{\mathrm{ND}}\right)},\theta =\frac{k_{2}}{\left(1+{\mathrm{BP}}_{\mathrm{ND}}\right)}$$

To solve equation (7) for a reduced acquisition time where $t=t_{s},t_{e}$, the basis function approach^15^ is used to pre-calculate the convolution term using the extrapolated $C_{R}(t=0,t_{e})$ with a range of biologically plausible values for θ. A least squares fit to the pre-determined data, $C_{T}^{d†}(t=t_{s},t_{e})$, is performed for each θ to estimate φ, and the instance of θ which yields the lowest sum of squares difference is selected. ${\mathrm{BP}}_{\mathrm{ND}}$ and *k*_2_ are then derived from φ, θ and the CBF-derived *R*_1_. For more details, including the pseudo-code, see Supplementary Materials.

### Data

Imaging data were collected from 60 cognitively normal subjects participating in Insight 46, a neuroimaging sub-study of the Medical Research Council National Survey of Health and Development,^19^ and 4 subjects from a study of young onset Alzheimer's Disease (YOAD) with an intermediate or high certainty diagnosis.^20^ These studies were conducted in line with the principles of the Declaration of Helsinki. For Insight 46, ethical approval was obtained from the National Research Ethics Service (ref 14/LO/1173), and for YOAD, approval was obtained from the London Queen Square Ethics Committee (ref 15/LO/1412). Written informed consent was obtained from all participants.

All subjects underwent 60 min of simultaneous amyloid PET and multi-modal MR imaging on a Siemens Biograph mMR PET/MR scanner. Of the 64 subjects used for analysis, mean age 69.6 years (range 61.7–70.5 years), 45 had both PET and ASL data, and for 19 the ASL data were missing, either due to repetition of other scans (4) or imaging artefacts (15).

**Figure 1.**
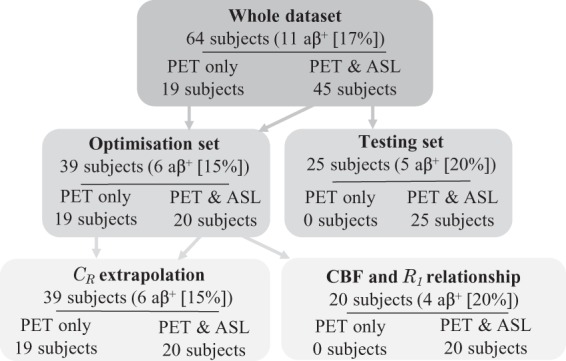
Flow chart describing data division between optimisation and testing sets, with the number of subjects defined as amyloid positive ($a\beta^{+}$) using SUVR with a whole cerebellum reference region.

The subjects were divided into 2 sets; an optimisation set containing 39 subjects, and a testing set containing the remaining 25 subjects, see Figure 1. The clinically diagnosed YOAD subjects were evenly split between the two sets, as were amyloid positive ($a\beta^{+}$) subjects from Insight 46. Amyloid positivity was defined using mean cortical grey matter SUVR with a whole cerebellum reference region, see Supplementary Materials for details.

Within the optimisation set, the 20 subjects with PET and ASL data were used to derive the relationship between PET-*R*_1_ and ASL-CBF. The whole optimisation set was used to optimise the extrapolation of *C_R_* and the acquisition timing window using leave-one-out cross validation. Finally, the relationship between PET-*R*_1_ and ASL-CBF and the optimised *C_R_* method from the optimisation set was used to apply the RT-SRTM to the 25 testing set subjects, see Supplementary Materials.

### CBF estimation from ASL MRI

CBF was estimated from a pseudo-continuous ASL (pCASL) acquisition with the following parameters: 3D GRASE readout^21^ with 36 partitions and a reconstructed voxel size of 1.88×1.88×4 mm, TE/TR=20.3/4000 ms, 4-shot with turbo-factor/EPI-factor=14/28, bandwidth 2298 Hz/pixel; 10 control-label pairs were acquired with a pulse duration (τ) and post labelling delay (PLD) both equal to 1800 ms. Acquisition time was 5 min 20 s (t=55,60). CBF maps were computed with equation (8) ^22^

(8) $$\mathrm{CBF}=\frac{6000\lambda }{2\alpha }\frac{\Delta S}{S_{0}}\frac{e^{\frac{\mathrm{PLD}}{T1_{\mathrm{blood}}}}}{T1_{\mathrm{blood}}(1-e^{-\tau /T1_{\mathrm{blood}}})}[\frac{\mathrm{ml}}{100g}/\text{min}]$$

with 0.9 ml/g for the plasma/tissue partition coefficient (λ), a blood T1 of 1650 ms ($T1_{\mathrm{blood}}$), and a labelling efficiency of 0.85 (α) as recommended in the ASL consensus paper.^23^ ΔS is the signal difference between the control and label images, *S*_0_ maps were estimated by fitting saturation recovery images acquired with the same sequence at three different saturation times (1,2,4 s) using NiftyFit.^24^

### Dynamic PET acquisition and reconstruction

List mode PET data were acquired for 60 min following intravenous injection of [^18^F]-florbetapir, which targets amyloid-β. For PET image reconstruction, simultaneously acquired structural T1- and T2-weighted MR images were used to synthesise CT data and calculate the attenuation map (μ-map),^25^ as validated in Ladefoged et al.^26^ The μ-map was propagated into PET space by registering the T1-weighted images to a full 60-min non-attenuation-corrected reconstructed PET image.

Dynamic PET data were binned into 31 time frames (15 s ×4, 30 s × 8, 60 s ×9, 180 s ×2, 300 s ×8), and reconstructed into 2×2×2 mm voxels using the open source NiftyPET package.^27^ An ordered subset expectation maximisation (OSEM) algorithm was used with 4 iterations, 14 subsets, and a 2 mm Gaussian filter, accounting for dead-time, attenuation, scatter, randoms and normalisation.

### Regional analysis

T1-weighted MR images were parcellated into 17 regions: accumbens, amygdala, brainstem, caudate, cerebellum (white and grey separately), hippocampus, cerebral white matter, pallidum, putamen, thalamus and six cortical grey matter regions, with left and right hemispheres combined;^28^ 16 regions were used for analysis, excluding the reference region.

Analysis was performed in native space such that the T1-weighted MR image was rigidly registered to both ASL (saturation recovery image target) and PET (µ-map transformation) space, and the transformation was propagated to the parcellation.^29^

### Statistical analysis

To compare different techniques with the gold standard, mean square error ($\mathrm{MSE}=\frac{1}{n}\sum_{i=1}^{n}(Y_{i}-Y_{i}^{*}{)}^{2}$ and mean error $(\mathrm{MSE}=\frac{1}{n}\sum_{i=1}^{n}(Y_{i}-Y_{i}^{*}))$ were used, where *n* is the number of estimates, $Y_{i}^{*}$ is the gold standard estimate or measured value and *Y_i_* is the estimate being evaluated. To compare different techniques, statistical significance was tested using paired, two-tailed Wilcoxon signed-rank test for MSE (as the data are not normally distributed), and paired two-tailed *t*-tests for ME.

## Results

### Relationship between ASL–CBF and PET-*R*_1_

**Figure 2.**
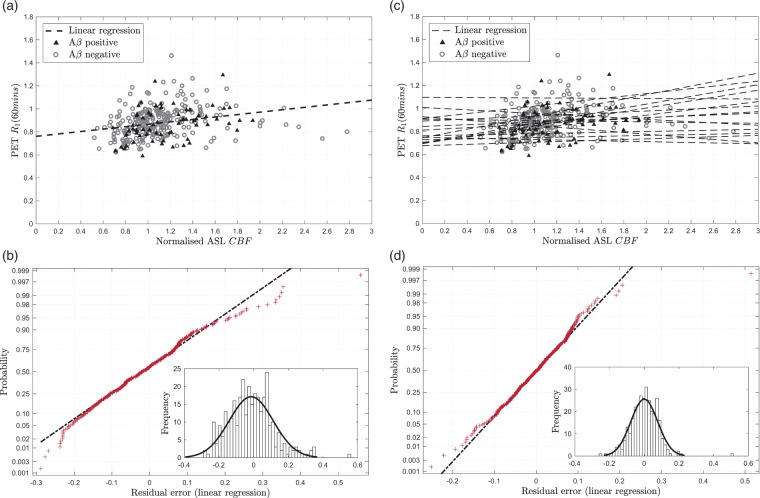
Correlation of PET-*R*_1_ with ASL-CBF for 20 optimisation set subjects where the regression was calculated and applied to the ASL-CBF data to show the residual error in the fit. (a) PET-*R*_1_ against ASL-CBF with single linear regression (black dashed line), (b) Residual normality plot for single linear regression *R*_1_ estimation, inset: histogram of residuals, (c) PET-*R*_1_ against ASL-CBF with multi-linear regression (black dashed lines), (d) Residual normality plot for multi-linear regression *R*_1_ estimation, inset: histogram of residuals.

Figure 2(a) shows the relationship between PET-*R*_1_ and ASL-CBF across the 16 regions for 20 optimisation set subjects. Linear regression shows a statistically significant correlation between the two parameters (ρ = 0.349, p< 0.001); however, there is some variability which is not explained by this linear model. Noise and artefacts in the ASL data are considered to be the main causes of the variability; however, violations of the model assumptions may also contribute.

Residual analysis was performed to determine whether a non-linear model could be fitted to the data, as suggested by equation (2). The normality plot for the residual error in Figure 2(a) is shown in Figure 2(b). This demonstrates that the residuals follow an approximately normal distribution, which supports the use of a linear model. However, there is some deviation from normality at the extremes, which is illustrated by the histogram inset in Figure 2(b). This shows that there are some outlying positive residuals which skew the distribution.

To quantify the regional and subject effects, multi-linear analysis was also performed. Multi-linear regression using the region name as a covariate was found to explain much of the variation seen in the single linear regression and gives an *R*^2^ value of 0.650 (adjusted *R*^2^ = 0.613). Figure 2(d) shows that the residual error using multi-linear regression is lower and more normally distributed than single linear regression.

Figure 2(c) shows the multi-linear regression by region which demonstrates the variability in slope and intercept between regions. These differences can be attributed to two main causes: regional differences in bolus transit times meaning that the ASL label image is acquired before the bolus reaches the tissue, and regional differences in tracer extraction. Acquiring ASL data with multiple post-labelling delay times can be used to reduce some of this variability and would be particularly helpful when transit time changes are caused by pathology which cannot be modelled. However, this multi-linear regression using region as a covariate provides a good model for the data used here.

To quantify the subject specific component of the relationship between normalised ASL-CBF and PET-*R*_1_ which cannot be modelled in a new set of subjects, multi-linear regression with the subject as a covariate was performed. This gave an *R*^2^ value of 0.436 (adjusted *R*^2^ = 0.358) indicating that there is some variation between subjects, but that this accounts for less of the variation than the variation between regions. Finally, multi-linear regression using both region and subject as covariates yielded an *R*^2^ value of 0.862 (adjusted *R*^2^ = 0.824), showing that most of the variation can be explained by these two parameters.

There is insufficient evidence that the data used here could support a more complex non-linear model, which supports the linear Renkin–Crone model in equation (2) under the assumption that $\frac{\mathrm{PS}}{f}\geq 3$. Since the multi-linear regression using region as a covariate is better able to describe the variation in the data, this model was selected for use when applying the RT-SRTM in the subsequent sections.

### Extrapolation of PET reference region, C_R_

#### Optimisation of PCA *C_R_* method

Table 1 shows the percentage of the variation described as the number of components are increased for the population of 39 subjects, $C_{R[0,60]}^{\mathrm{pop}}$. This demonstrates that six principal components are required to describe 99.9% of the variation within the data; therefore, a maximum of six components are used in the optimisation (L≤6).

**Table 1.** The percentage of the variation explained using increasing number of principal components following PCA on the $C_{R[t=0,60]}$ of 39 optimisation set subjects.

Number of components (*L*)	Variation described (%)
1	76.4
2	95.0
3	97.8
4	99.1
5	99.6
6	99.9

**Figure 3.**
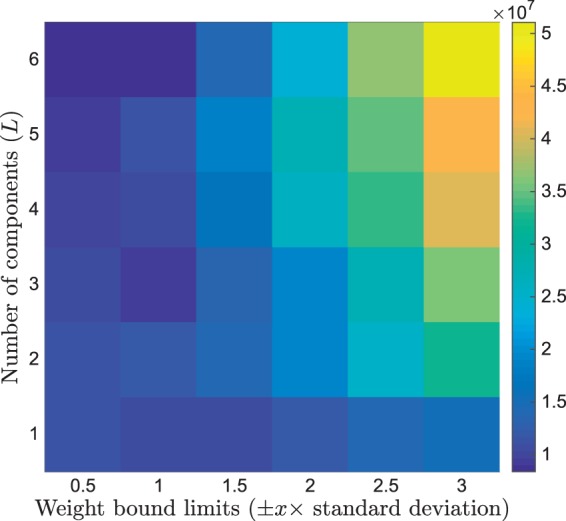
MSE in the fit of $C_{R[t=30,60]}$ using the *PCA* *C_R_* method when optimising the number of components used and the upper and lower bounds for the weights using leave-one-out analysis on 39 optimisation set subjects.

Figure 3 shows the MSE in the fit of $C_{R[t=30,60]}$ using leave-one-out cross validation and averaged across subjects. This demonstrates that constraining the upper and lower bounds of the weights reduces the error when fitting to data with missing timepoints. The number of components used (*L*) has less of an influence on the error, and overall the combinations of components and weight bounds with the lowest error are L=6 with either ± 0.5 or 1 times the standard deviation for the bounds, or *L* = 3 with ± 1 times the standard deviation for the bounds. These three combinations give a similar MSE for t=30,60; however, L=6 with 1 times the standard deviation for the bounds was found to perform consistently better across different timing windows, and therefore this was used for comparison with the *scaled mean* *C_R_* method in the following sections.

#### Comparison of PCA *C_R_* and scaled mean *C_R_* methods

**Figure 4.**
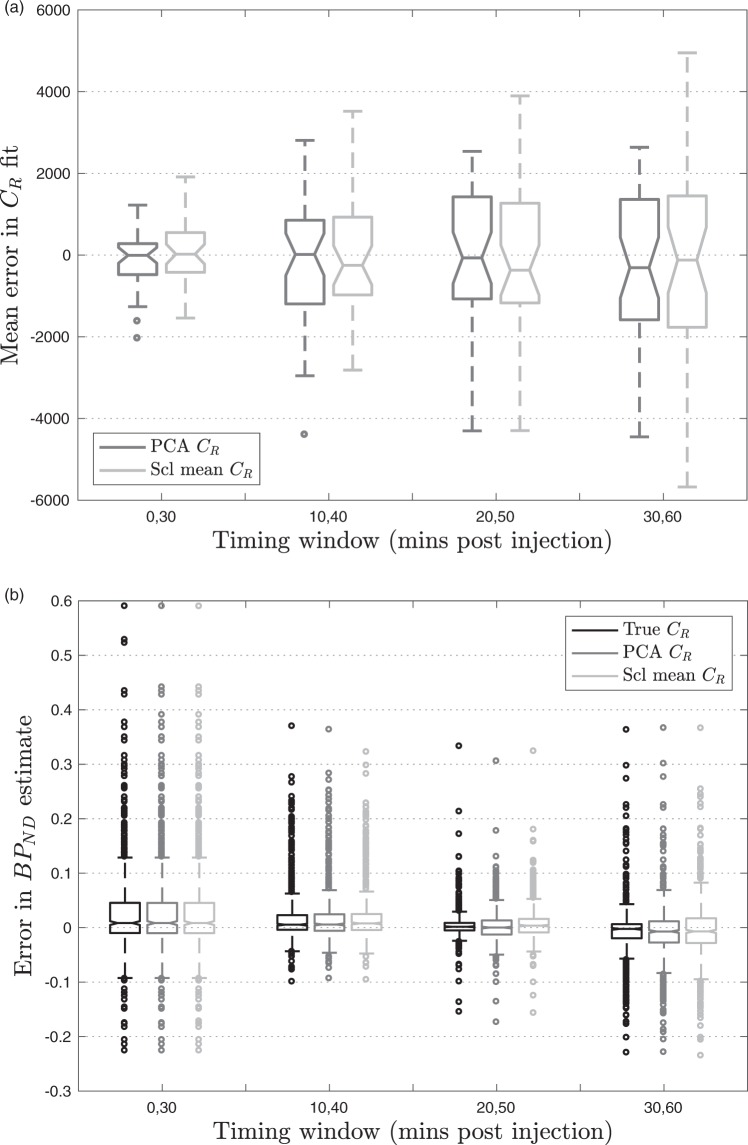
Box-plots calculated using leave-one-out cross validation in 39 optimisation set subjects. (a) ME in the *PCA* *C_R_* method and *scaled mean* *C_R_* method compared to measured *C_R_*, (b) Error in ${\mathrm{BP}}_{\mathrm{ND}}$ estimates across different timing windows using different estimates of *C_R_*.

The boxplot in Figure 4(a), which summarises across all subjects, shows that the *PCA* *C_R_* method performs better for t=0,30 and t=20,50; however, the difference in MSE did not reach statistical significance at any timepoint (p≥0.241). The influence of this error on the estimation of ${\mathrm{BP}}_{\mathrm{ND}}$ at different acquisition windows is assessed in the next section.

### Optimisation of data acquisition window

The MSE and ME in the estimation of ${\mathrm{BP}}_{\mathrm{ND}}$ using different data acquisition windows are shown in Table 2. Extrapolation of *C_R_* is not strictly necessary for t = 0,30 as it starts from injection; thus, the basis functions can be generated using the measured data alone. However, this leads to a higher MSE of 0.1960 due to higher errors in the computation of the convolution, and therefore the results given always extrapolate *C_R_* for t = 0,30.

**Table 2.** MSE and ME between gold standard ${\mathrm{BP}}_{\mathrm{ND}}^{*}$ and ${\mathrm{BP}}_{\mathrm{ND}}$ at different 30-min acquisition windows averaged across 16 regions and 39 optimisation set subjects.

Time window (*t= t_s_, t_e_*)						True *C_R_*		PCA *C_R_*		Mean *C_R_*	
0,10	10,20	20,30	30,40	40,50	50,60	MSE	ME	MSE	ME	MSE	ME
•	•	•	◯	◯	◯	0.0089	0.0303	0.0085	0.0299	0.0085	0.0299
◯	•	•	•	◯	◯	0.0035	0.0202	0.0036	0.0202	0.0035	0.0211
◯	◯	•	•	•	◯	**0.0008**	** 0.0050**	** 0.0010**	** 0.0041**	** 0.0012**	0.0088
◯	◯	◯	•	•	•	0.0030	–0.0084	0.0032	–0.0090	0.0034	**–0.0050**

Note: True *C_R_* uses the true reference region curve, PCA *C_R_* and mean *C_R_* extrapolate the reference region curve using the *PCA* *C_R_*and *scaled mean* *C_R_* methods, respectively. All methods used the gold standard $R_{1}^{*}$.

In Table 2, the true *C_R_* column uses the full measured $C_{R}(t=0,60)$, therefore errors are introduced purely due to the limited number of datapoints available. When the later frames are omitted and only t = 0,30 min of data are acquired, large errors are introduced as there is little information about the late phase which contains the signal relating to target binding. As the time window is shifted later, the MSE and ME are reduced.

However, the results in Table 2 also show that there is little to be gained by including data acquired more than 50 min post injection as the error increases. This is because the signal has plateaued by this point so, for a fixed 30-minute window, a better fit can be obtained by including some of the earlier data where the tracer concentration changes more rapidly over time. This is further illustrated in Figure 4(b), which shows the smallest distribution of errors in ${\mathrm{BP}}_{\mathrm{ND}}$ at t = 20,50.

For the two techniques which extrapolate *C_R_* from the available time window, additional variability is introduced into the ${\mathrm{BP}}_{\mathrm{ND}}$ estimates due to errors in the extrapolation, which is reflected by the higher MSE in Table 2. For both the *PCA* *C_R_* and the *scaled mean* *C_R_* method, the early phase of the data becomes more important, as much of the variation between the *C_R_* of subjects is contained in these frames. This can be seen in Figure 4(a), as for t = 0,30 the error in *C_R_* estimation is at a minimum and increases as the acquisition window shifts later. When the early frames are included, the error in ${\mathrm{BP}}_{\mathrm{ND}}$ is very similar to using the true *C_R_*, whereas when only the late frames are used, the error in ${\mathrm{BP}}_{\mathrm{ND}}$ for the extrapolation methods increases relative to the true *C_R_*, Figure 4(b). Table 2 shows that the optimal timing window for this technique is t = 20,50 min post injection as it yields the lowest MSE and ME.

The *PCA* *C_R_* method produces a consistently lower MSE which was statistically significantly lower than that obtained using the *scaled mean* *C_R_* method for t = 20,50 (*p* = 0.002). This is due to the increased flexibility in this method which allows it to better describe the variation in unseen *C_R_* shapes. The *PCA* *C_R_* method also produces ${\mathrm{BP}}_{\mathrm{ND}}$ estimates with lower bias at t = 20,50, as demonstrated by the median lines in Figure 4(b) and the ME in Table 2 (*p* < 0.001).

Due to the lower MSE and ME, the *PCA* *C_R_* method with the t = 20,50 min timing window was selected for the full implementation of the RT-SRTM in subsequent sections.

### Comparison of proposed RT-SRTM with gold standard

**Figure 5.**
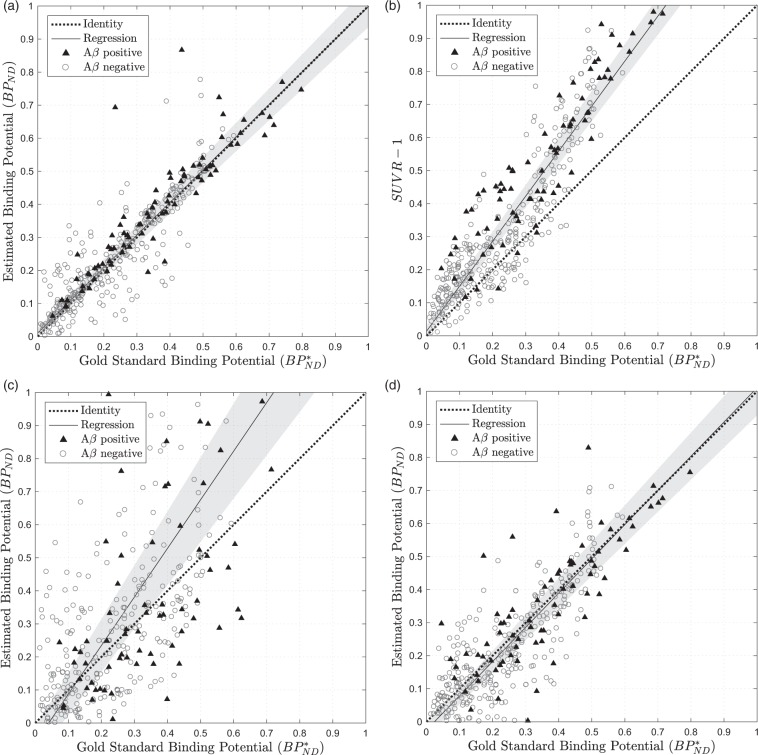
Estimated amyloid burden against the gold standard value calculated using full PET time series for 25 testing set subjects using 4 different methods: (a) RT-SRTM where t = 20,50 min (ASL-derived *R*_1_), (b) SUVR-1 where t = 50,60 min, (c) SRTM where t = 0,30 min (PET data only, no *C_R_* extrapolation), (d) RT-SRTM where t = 20,50 min (PET data only, extrapolated *C_R_*). The grey-shaded region covers the 95% confidence interval in the regression.

Figure 5(a) shows ${\mathrm{BP}}_{\mathrm{ND}}$, estimated using the RT-SRTM with t = 20,50 min of data plotted against the gold standard ${\mathrm{BP}}_{\mathrm{ND}}^{*}$. Linear regression of the data shows that the RT-SRTM method offers a good approximation of the gold standard as it closely follows the line of identity (dashed), which is within the 95% confidence interval (CI) of the regression (shaded). The linear correlation between the two estimates was tested using the Pearson correlation coefficient which demonstrated a high, statistically significant result (ρ = 0.896, *p* < 0.001, 95% CI [0.875, 0.914]).

To determine the influence of CBF derived *R*_1_ errors on ${\mathrm{BP}}_{\mathrm{ND}}$, sensitivity analysis was performed as described in the supplementary materials. Briefly, PET time activity curves were simulated using the realistic parameters with a range of noise values^30^ and fitted using the optimised RT-SRTM with a fixed *R*_1_ error. It was found that, for regional analysis where noise < 3.4%, the mean absolute error was smaller than the blood flow component found in SUVR in Cselenyi et al.^4^ However, if the methodology were to be extended to voxel-wise analysis, a lower *R*_1_ error would be required due to the increased noise in the PET data which increases the uncertainty in the model fit.

### Comparison of proposed RT-SRTM with SUVR-1

To ensure a fair comparison between RT-SRTM and SUVR, SUVR-1 was calculated at five 10-minute acquisition windows, starting at t = 30, to cover the recommended time range and compared to the gold standard ${\mathrm{BP}}_{\mathrm{ND}}$, see supplementary materials. The optimal timing window with the lowest bias and error was found to be t = 50,60 minutes post injection, which is in concordance with the literature,^4^ hence t = 50,60 was used for comparison.

Figure 5(b) shows the amyloid burden estimates generated for SUVR-1 (t = 50,60 minutes). Whilst the correlation between SUVR-1 and ${\mathrm{BP}}_{\mathrm{ND}}^{*}$ is evident, a positive bias is shown as SUVR-1 overestimates the binding potential at higher values. This is due to the fact that the target and reference tissue concentrations reach equilibrium with blood plasma at different points depending on tracer binding, as has been explored in detail in the literature.^31^ The ME quantifies the bias between the estimates and the gold standard which is 0.1038 for SUVR-1, indicative of the systematic overestimation, compared to 0.0079 for the RT-SRTM method (*p* < 0.001). The RT-SRTM method also has a lower MSE (0.0066 compared to 0.0235 for SUVR-1, *p* < 0.001), showing that overall this technique is more accurate at estimating ${\mathrm{BP}}_{\mathrm{ND}}^{*}$ than the simplified technique.

This overestimation in SUVR-1 is likely to be a combination of a linear systematic error in the estimation, as well as the influence of blood flow. Systematic error could result from estimating SUVR-1 when not at steady-state, and could potentially be accounted for using a population correction factor.

To determine the influence of blood flow on the estimation of target density, the correlation between estimated target density and tracer delivery was calculated, as in Cselényi and Farde^4^ Spearman's correlation (ρ) between $R_{1}^{*}$ and the estimates was calculated, as the relationship is in theory non-linear. For the gold standard ${\mathrm{BP}}_{\mathrm{ND}}^{*}$ and ${\mathrm{BP}}_{\mathrm{ND}}$ using RT-SRTM, there was no significant correlation with $R_{1}^{*}$ (*p* = 0.336 and 0.106 respectively). However, for SUVR-1, there was a significant negative correlation (ρ = −0.226, *p* < 0.001), which suggests that the proposed RT-SRTM method may be more robust to changes in blood flow than SUVR-1.

### Comparison of the proposed RT-SRTM with short acquisition time PET

Pharmacokinetic modelling can be applied to reduced acquisition time PET data without incorporating ASL data in several ways, e.g.: (i) fitting the first 30 min of PET data only using the standard SRTM in equation (1), (ii) extrapolating *C_R_* as in RT-SRTM, but *R*_1_ is estimated from the PET data.

For the first method, (i), Figure 5(c) compares the estimation of ${\mathrm{BP}}_{\mathrm{ND}}$ using the PET data for t = 0,30 min to the gold standard. It is evident that the absence of late-phase data leads to a high error in the estimate, which is significantly higher than using the RT-SRTM (MSE = 0.0813, *p* < 0.001; ME = 0.0569, *p* < 0.001).

Using the second method, the estimation of ${\mathrm{BP}}_{\mathrm{ND}}$ using t = 20,50 min of PET data and extrapolating using the *PCA* *C_R_* method, Figure 5(d) shows a significantly lower MSE compared to using the first 30 min (MSE = 0.0121, *p* < 0.001). The timing window t = 20,50 was selected by calculating the MSE for all the timing windows used in Table 2 and t = 20,50 yielded the lowest value. This method also outperforms SUVR-1 with a lower MSE (*p* < 0.001) and ME (*p* < 0.001); however, it is significantly correlated with the gold standard $R_{1}^{*}$ (*p* = 0.004).

Comparison of the PET only method with the proposed RT-SRTM including ASL derived *R*_1_ estimates shows that the additional CBF information improves the estimate of ${\mathrm{BP}}_{\mathrm{ND}}$, yielding a significantly lower MSE (*p* = 0.028), ME (*p* < 0.001) and variance (two-tailed F-test *p* < 0.001). This can be seen in Figure 5(a) where the points are more tightly clustered around the line of identity when using the proposed RT-SRTM compared to Figure 5(d) using PET data only.

## Discussion

In this paper, we have presented an improved framework for quantitative PET analysis with significantly reduced acquisition time, exploiting blood flow information from simultaneously acquired ASL MRI data.

We have demonstrated that the relationship between the blood flow and tracer delivery for [^18^F]-florbetapir, which is described by the Renkin–Crone model, may be approximated as regionally linear and used to convert ASL relative CBF values into pseudo-*R*_1_ estimates. We have also evaluated a new technique for extrapolating the reference region time activity curve, *C_R_*, using PCA which introduces a lower error than the method used by Scott et al.,^9^ where the mean population is scaled. The timing of the PET acquisition was then optimised, and found to be t = 20,50 min post injection.

When the RT-SRTM estimates of ${\mathrm{BP}}_{\mathrm{ND}}$ using t = 20,50 min of PET data and ASL derived *R*_1_ were compared to the gold standard using the full 60 min of PET data, a strong linear correlation was found. This demonstrates that the RT-SRTM with a 30-min acquisition could potentially be used as a proxy for the full 60-min acquisition for this tracer and subject group.

By comparison, the simplified measure, SUVR-1, using 10 min of data showed a strong positive bias in the target density estimation, and the results were correlated with the delivery of the tracer as determined by the gold standard *R*_1_ estimates. This implies that, in addition to systematic error within the SUVR-1 estimates, there is also a bias introduced due to local differences in blood flow. This may confound longitudinal studies, as blood flow may change over time, over the progression of disease, or due to disease modifying interventions. Conversely, the RT-SRTM estimates of target density were not correlated with *R*_1_, suggesting that this technique may be robust to changes in blood flow and could be a suitable alternative for longitudinal studies. However, this needs to be validated in a longitudinal dataset.

SUVR-1 estimation at different timing windows showed that t = 50,60 min gave the best estimation of ${\mathrm{BP}}_{\mathrm{ND}}$. Since SUVR-1 appears to have plateaued by this point, it is unlikely that the estimation can be improved by acquiring data at a later timepoint.

This paper focused on the optimisation of a 30-min PET/MR acquisition. Simultaneous acquisition ensures that the CBF measured by ASL represents the flow at tracer injection, avoiding errors introduced by physiological flow changes throughout the day.^2^ This assumes negligible change in blood flow between the tracer injection and the end of the scan. This can be controlled through measures used for routine clinical PET scans, such as keeping the patient lying down in an uptake room from injection to scan start. The influence of auditory stimulation, which would be present during the ASL scan but not during tracer injection, on CBF should also be considered, and if necessary the conditions in the scanner should be emulated in the uptake bay. If conditions could be adequately controlled, the technique could be extended to separate PET and MRI acquisitions. However, this would be heavily dependent on the scheduling of the scans and the acquisition time saved is reduced compared to the simultaneous PET/MRI method.

A 30-min acquisition was selected to accommodate a typical MRI neuroimaging session, while still greatly increasing patient throughput and comfort. This could be further reduced depending on the MRI data acquired, where the minimum time is determined by the acquisition of the ASL data and the images required for attenuation correction of the PET data. In this case, using the *PCA* *C_R_* method with fewer principal components should be considered to avoid an under-determined problem where there are more parameters to fit than datapoints available.

The ASL data used in this study were acquired for just 5.5 min over 50 min into the scan with no motion correction and no patient restraint. For this reason, approximately 30% of the ASL-CBF maps failed quality control checks, largely due to motion-induced artefacts. This represents a challenging dataset which could be significantly improved by increasing the number of acquisitions and motion correction. However, the fact that the RT-SRTM worked so well on this dataset indicates that it could be a clinically useful tool. It is worth noting that the data acquired from the four clinically diagnosed YOAD subjects all passed the quality control checks.

The limited time available within the protocol for the ASL acquisition also meant that a single delay time between blood tagging and image acquisition was used. This yields errors in CBF estimation due to different bolus transit times for different brain regions, either due to normal physiology or pathological changes. A multi-delay time ASL acquisition would make the methodology more robust as the transit time is parameterised within the model and this will be evaluated in future work.

The RT-SRTM was here applied to an amyloid-β tracer; however, the methodology could potentially be used for any tracer which can be described by the SRTM, and which has a sufficiently high extraction fraction, such as [^18^F]-flutametamol, another amyloid-β tracer, or tau (τ) tracers.^32^ The kinetics of these tracers are slower than those of [^18^F]-florbetapir, and as such require longer dynamic acquisitions. Here, the RT-SRTM could potentially offer a greater reduction in acquisition time, and in future work, we intend a comparison with the dual time-window protocol, another acquisition time reduction method used on such tracers, where early and late PET data are acquired with a break in-between.^33^ Furthermore, our approach could be broadened to other kinetic models which have a tracer delivery parameter that can be approximated using CBF from ASL. Reference region curve extrapolation could also be used in reference Logan analysis,^34^ as an alternative to a previously proposed reduced acquisition time method which cannot account for blood flow changes.^35^

The main limitation of this study is that the optimisation of the RT-SRTM has been performed on cross-sectional dynamic scans of mostly healthy volunteers. The introduction of subjects with disease may increase the variability in *C_R_* between subjects. However, some variability already exists in the dataset used as the healthy subjects undergo normal ageing and we include four clinically diagnosed subjects, which the *PCA* *C_R_* method can handle. Provided that the dataset used to build the model for the *PCA* *C_R_* method includes diseased subjects, this variability can also be accounted for.

In future work, this technique will be validated on longitudinal data including symptomatic patients, to further investigate its robustness to disease progression and local changes in blood flow. The linear relationship found between *R*_1_ and relative CBF will require verification on such datasets to ensure that the assumption of $\frac{\mathrm{PS}}{F}\geq 3$ still holds in symptomatic subjects.

Another limitation within the proposed technique is that tracer delivery is estimated directly from ASL-CBF using a region-dependent linear relationship. This works well for regional data as artefacts can be averaged out; however, this is not possible for voxel-wise analysis where errors will propagate through to the *R*_1_ estimate. A more robust methodology which propagates database *R*_1_ values into the subject space based on local image similarity has been proposed^36^ and will be investigated in the future. This would facilitate voxel-wise analysis for quantitative parametric imaging within a clinically feasible time frame.

## Supplemental Material

Supplemental material for Reduced acquisition time PET pharmacokinetic modelling using simultaneous ASL–MRI: proof of concept Click here for additional data file. Supplemental material for Reduced acquisition time PET pharmacokinetic modelling using simultaneous ASL–MRI: proof of concept by Catherine J Scott, Jieqing Jiao, Andrew Melbourne, Ninon Burgos, David M Cash, Enrico De Vita, Pawel J Markiewicz, Antoinette O'Connor, David L Thomas, Philip SJ Weston, Jonathan M Schott, Brian F Hutton and Sébastien Ourselin in Journal of Cerebral Blood Flow & Metabolism
