# Supplementary material for: Reduced acquisition time PET pharmacokinetic modelling using simultaneous ASL–MRI: proof of concept
Source: J Cereb Blood Flow Metab. 2018 Sep 5;39(12):2419–32. doi: 10.1177/0271678X18797343 (PMC6891000; doi:10.1177/0271678X18797343)
Supplement: Supplemental material for Reduced acquisition time PET pharmacokinetic modelling using simultaneous ASL–MRI: proof of concept [file Supplemental_material_6.pdf]

SUPPLEMENTARY INFORMATION

Title

Spatiotemporal analysis of impaired microglia process movement at sites of secondary neurodegeneration post-stroke.

Author names

Murielle G. Kluge[1, 2]; Mahmoud Abdolhoseini[3]; Katarzyna Zalewska[1,2] , Lin Kooi Ong[1, 2, 4]; Sarah J. Johnson[3]; Michael Nilsson [2, 4#]; Frederick R. Walker[1, 2, 4#*]

Affiliations

[1] School of Biomedical Sciences and Pharmacy and the Priority Research Centre for Stroke and Brain Injury, University of Newcastle, Callaghan, NSW, Australia.
[2] Hunter Medical Research Institute, Newcastle, NSW, Australia.
[3] School of Electrical Engineering and Computer Science, University of Newcastle, Callaghan, NSW, Australia.
[4] NHMRC Centre of Research Excellence Stroke Rehabilitation and Brain Recovery, Heidelberg, VIC, Australia.
[#]contributed equally as senior authors

Corresponding author

Frederick R. Walker

School of Biomedical Sciences and Pharmacy and the Priority Research Centre for Stroke and Brain Injury, University of Newcastle, Callaghan, NSW, Australia. Hunter Medical Research Institute, Newcastle, NSW, Australia.

Phone: +61 492 15012

E-mail: rohan.walker@newcastle.edu.au

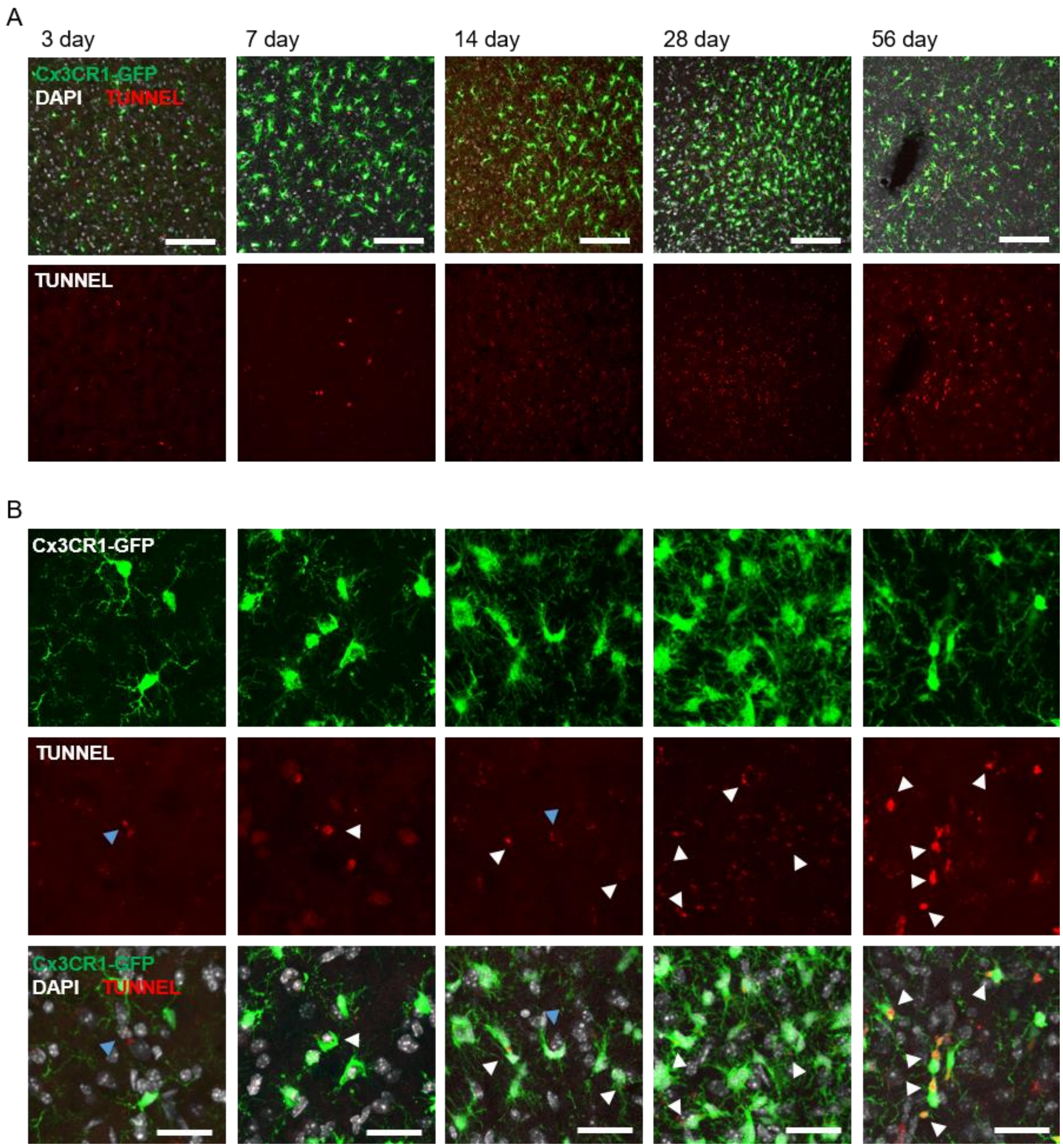

Supplementary Figure 1: Click iT TUNEL assay for apoptotic cell in the ipsilateral thalamus over time after stroke.

A) Representative maximum projection confocal images of the Po 3, 7 14, 28, 56 days after stroke. Fixed brain slices of Cx3CR1$^{GFP/WT}$ mice expressing GFP- label microglia (green) were co-labelled with Click iT TUNEL staining for DNA fragmentation (red and bottom panel) and DAPI (white). Scale bar, 100 µm.

B) Representative single cell, high magnification maximum projection images of images shown in A). Nuclei stained for both TUNEL and DAPI appear internalized by GFP labeled microglia (white arrowheads). Non-internalised TUNEL positive cells are indicated by blue arrowheads. Merge in bottom panel (TUNEL: red; GFP: green; DAPI: white). Scale bar, 25 µm.

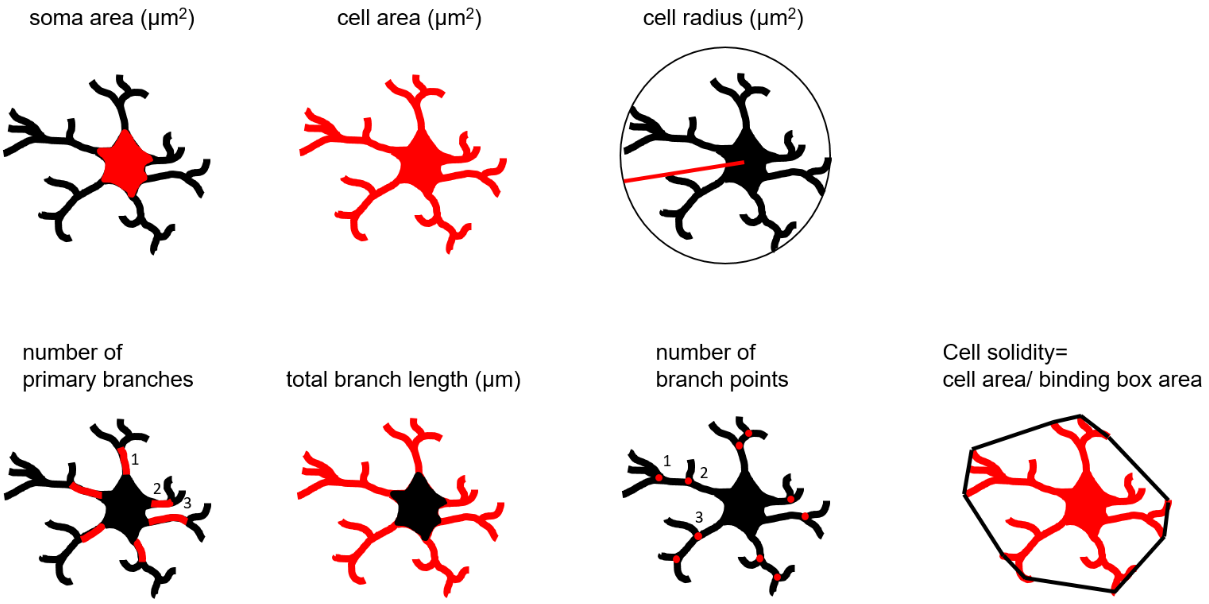

Supplementary Figure 2: Schematic illustration of parameters assessed for morphological analysis in 'MicroTrac'.

Red parts of the cell indicate the measured parameters. Please note: cell solidity is calculated as cell area divided by the binding box area, cell radius is calculated from the centre of the cell to the end of the longest branch.

Supplementary movies legends

All supplementary movies are imaged *ex-vivo* using multi-photon imaging of acute brain slices from Cx3CR1$^{GFP/WT}$ mice. Laser injuries were induced by scanning the central z-plane at a zoom factor of 48x for 1sec. One baseline z-stack was taken pre-ablation and then subsequently every 1 min for 15 min post ablation. Maximum projection images were generated and rectified in the x-y plane.

Supplementary movie 1: Microglia response to a laser ablation within the ipsilateral **thalamus** (Posterior complex) **3 days** after a cortical photothrombotic stroke (.avi 1.71MB).

Supplementary movie 2: Microglia response to a laser ablation within the ipsilateral **thalamus** (Posterior complex) **7 days** after a cortical photothrombotic stroke (.avi 1.63 MB).

Supplementary movie 3: Microglia response to a laser ablation within the ipsilateral **thalamus** (Posterior complex) **56 days** after a cortical photothrombotic stroke (.avi 1.94 MB).

Supplementary movie 4: Microglia response to a laser ablation within the **peri-infarct** territory **3 days** after a cortical photothrombotic stroke (.avi 2.03 MB).
